# Supplementary material for: Crypto-coding technique based on polar code and secret key generated from wireless channel characteristics for wireless communication systems
Source: PLoS One. 2025 Feb 25;20(2):e0318110. doi: 10.1371/journal.pone.0318110 (PMC12072636; doi:10.1371/journal.pone.0318110)
Supplement: S1 Fig — (PDF) S2 Fig. Secret key generation steps for the wireless communication systems. (PDF) S3 Fig. The secure transmission model based on the polar code. (PDF) S4 Fig. Proposed crypto-coding scheme based on polar code. (PDF) S5 Fig. An example of the proposed secure polar code. (PDF) S6 Fig. The BER of the systems with conventional polar code and the proposed secure polar code in case of N = 1024 and modified R. (PDF) S7 Fig. The BER of the systems with conventional polar code and the proposed secure polar code in case of N = 2048 and modified R. (PDF) S8 Fig. The BER of the systems with conventional polar code and the proposed secure polar code in case of R = 1 ∕ 4 and modified N. (PDF) S9 Fig. The BER of the systems with the conventional polar code and the proposed secure polar code in case of R = 1 ∕ 8 and modified N. (PDF) S10 Fig. Security performance for N = 128, R = 1 ∕ 2. (PDF) S11 Fig. Security performance for N = 256, R = 1 ∕ 4. (PDF) S1 Table. Parameters for some NIST tests. (PDF) S2 Table. The tested values of the required NIST tests. (PDF) S3 Table. Simulation parameters. (PDF) S4 Table. Comparing our method with the previous methods for N = 1024 and R = 1/2. (PDF) S5 Table. CComparing the security performance of the proposed method andthe previous methods. (PDF) S6 Table. Total time complexity of the conventional polar code and the proposed secure polar code with R = 1 ∕ 2. (PDF) [file pone.0318110.s001.pdf]

Supporting Information

S1 Fig. The system model.

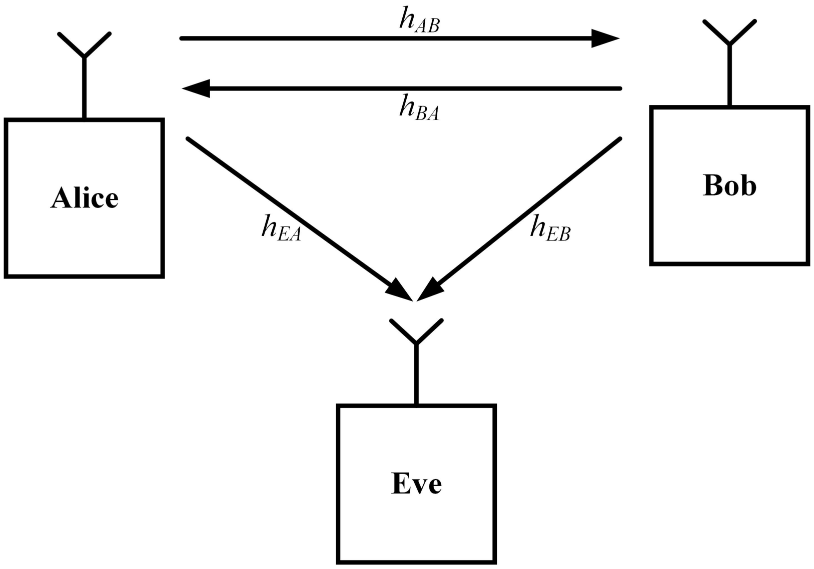

S 2 Fig. Secret key generation steps for the wireless communication systems.

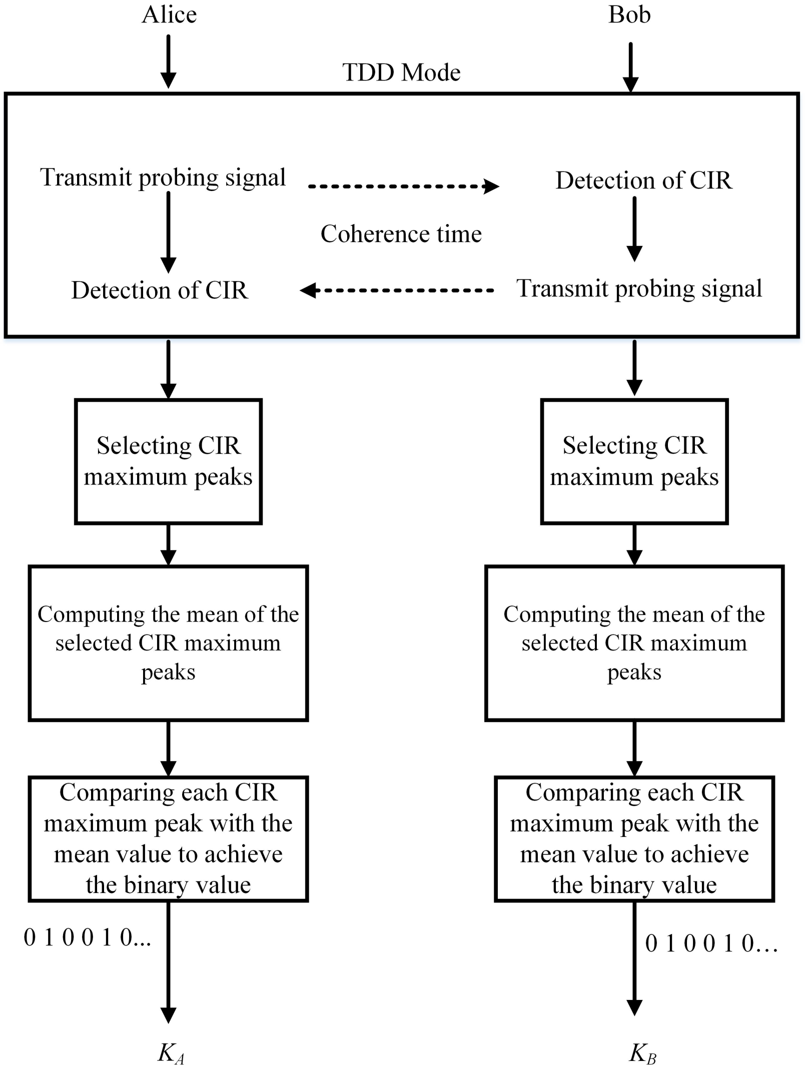

S 3 Fig. The secure transmission model based on the polar code.

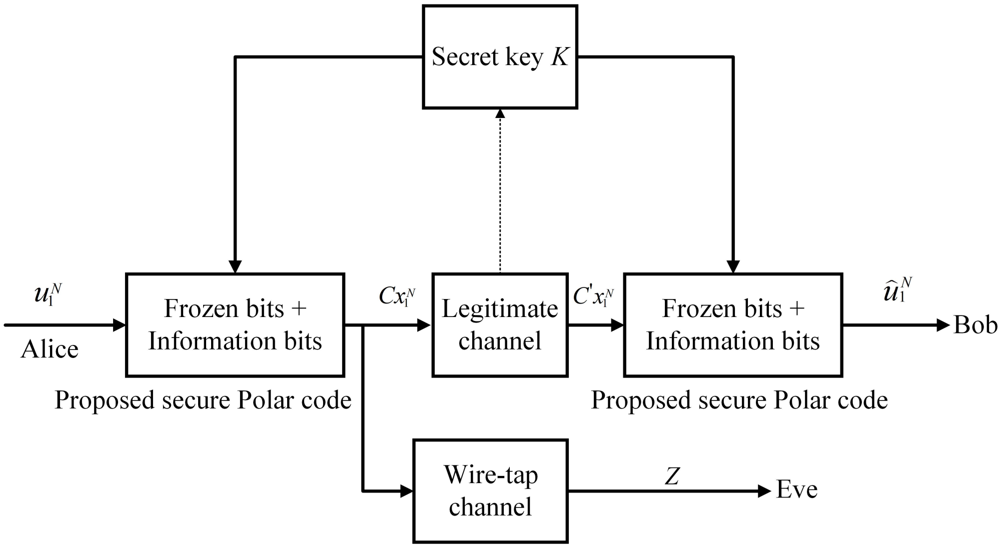

S 4 Fig. Proposed crypto-coding scheme based on polar code.

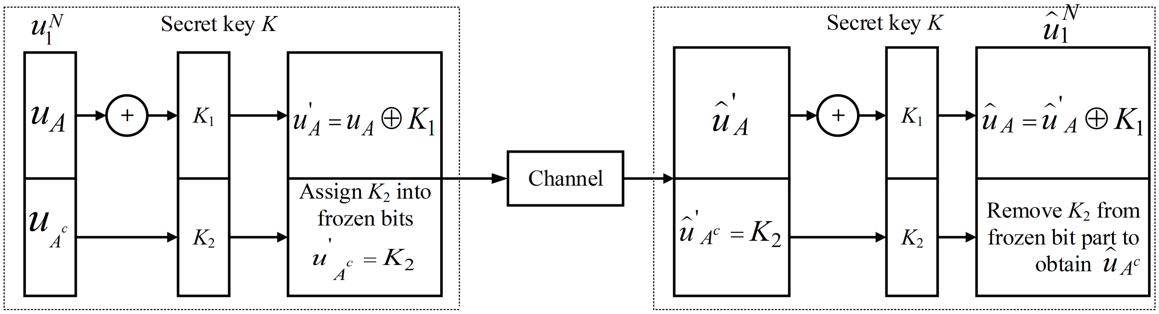

S 5 Fig. An example of the proposed secure polar code.

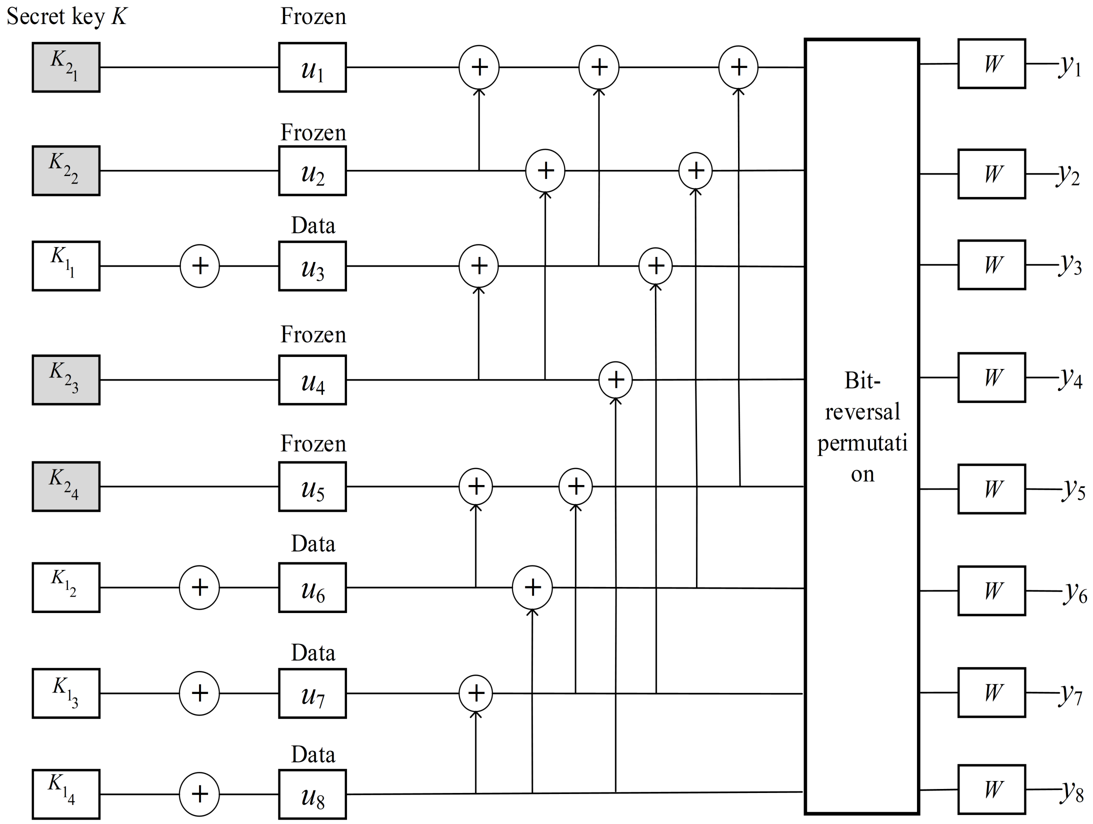

S 6 Fig. The BER of the systems with conventional polar code and the proposed secure polar code in case of $N = 1024$ and modified R.

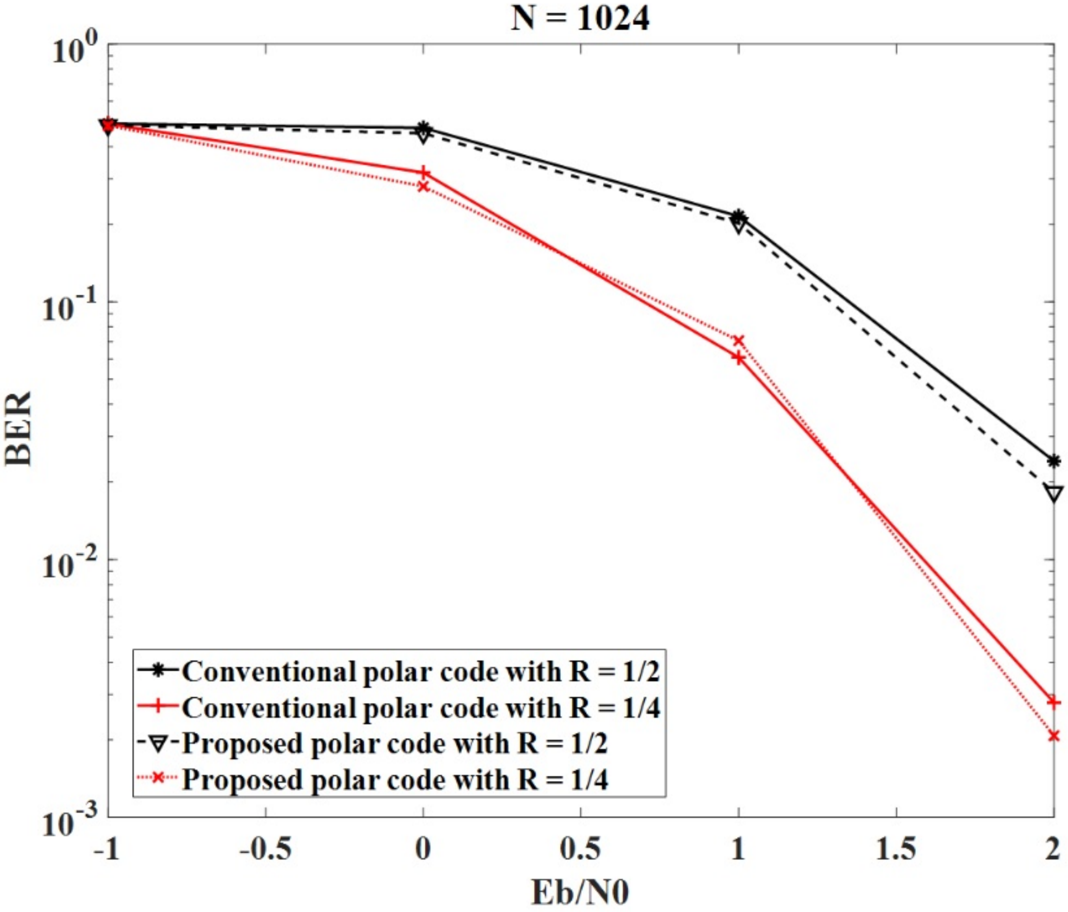

S 7 Fig. The BER of the systems with conventional polar code and the proposed secure polar code in case of $N = 2048$ and modified R.

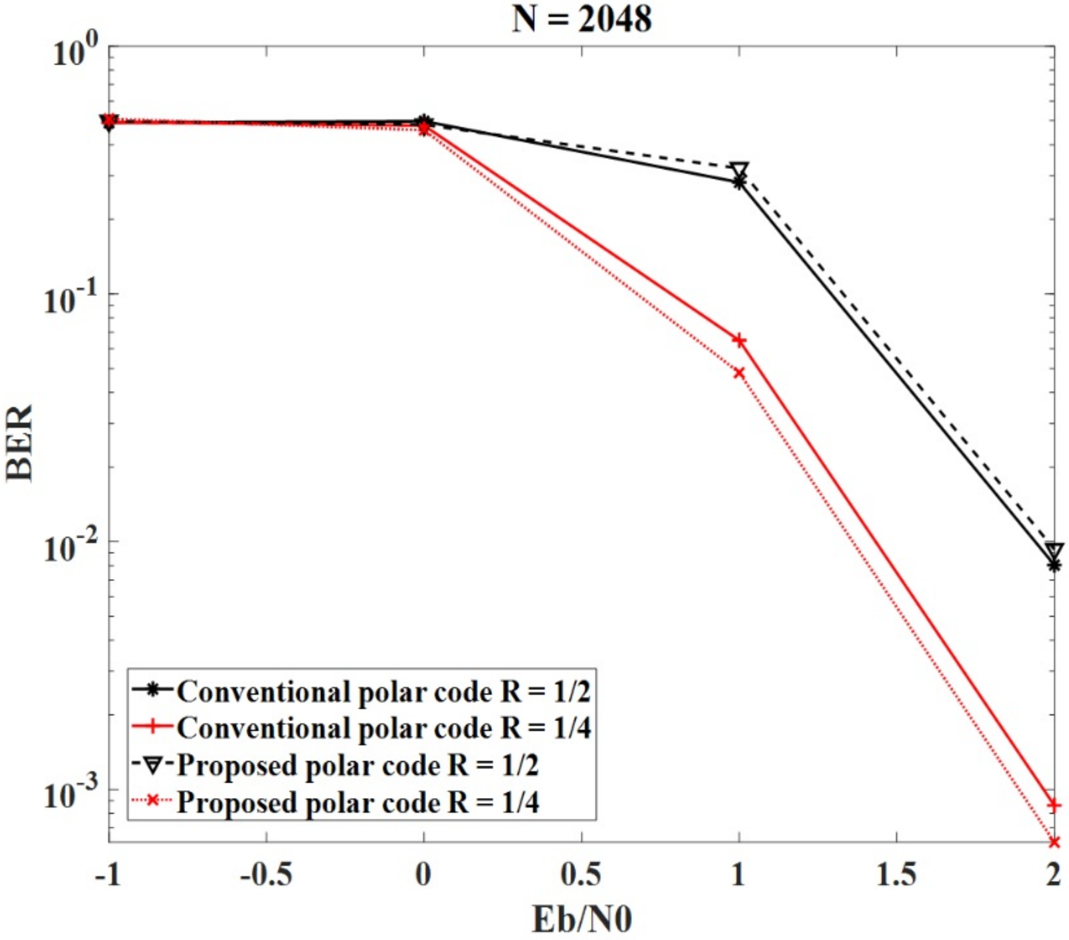

S 8 Fig. The BER of the systems with conventional polar code and the proposed secure polar code in case of $R = 1/4$ and modified N.

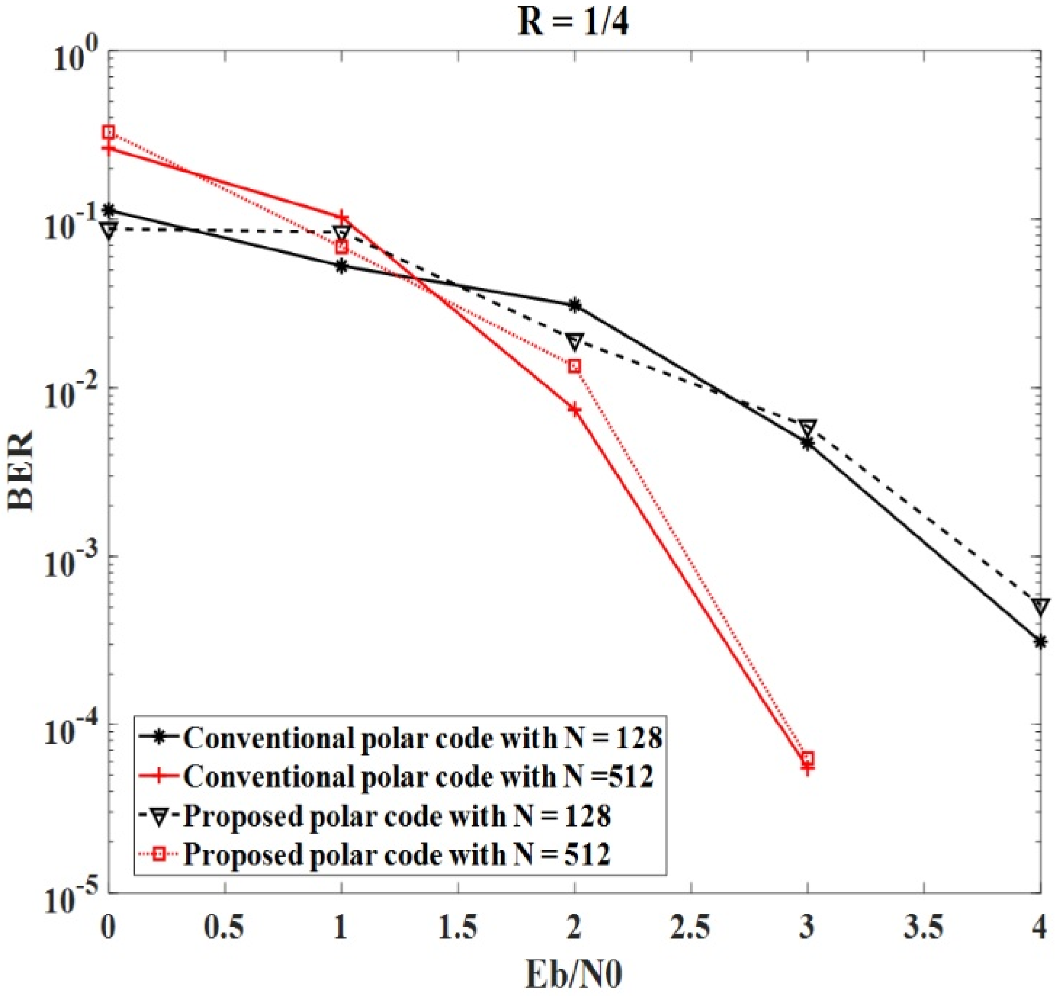

S 9 Fig. The BER of the systems with the conventional polar code and the proposed secure polar code in case of $R = 1/8$ and modified N.

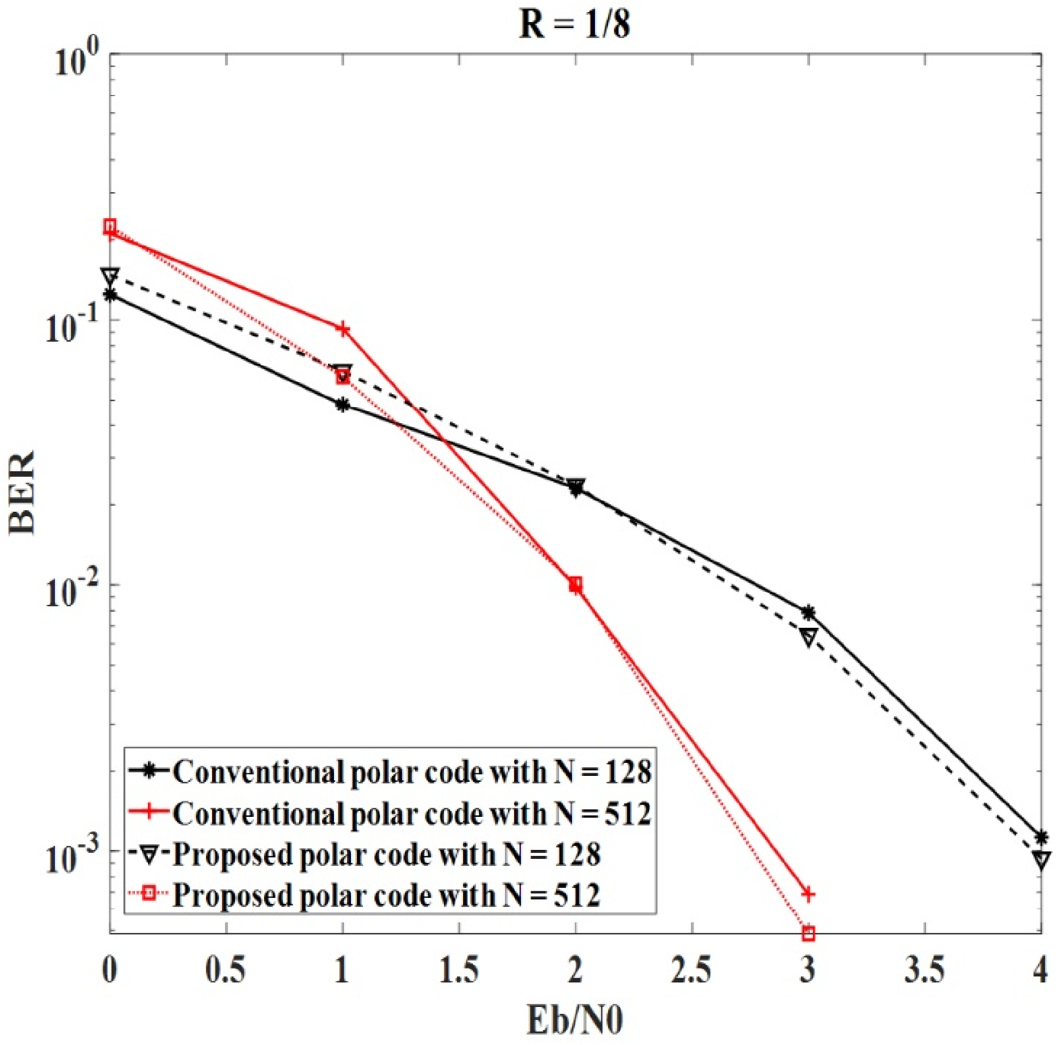

S 10 Fig. Security performance for $N = 128$, $R = 1/2$.

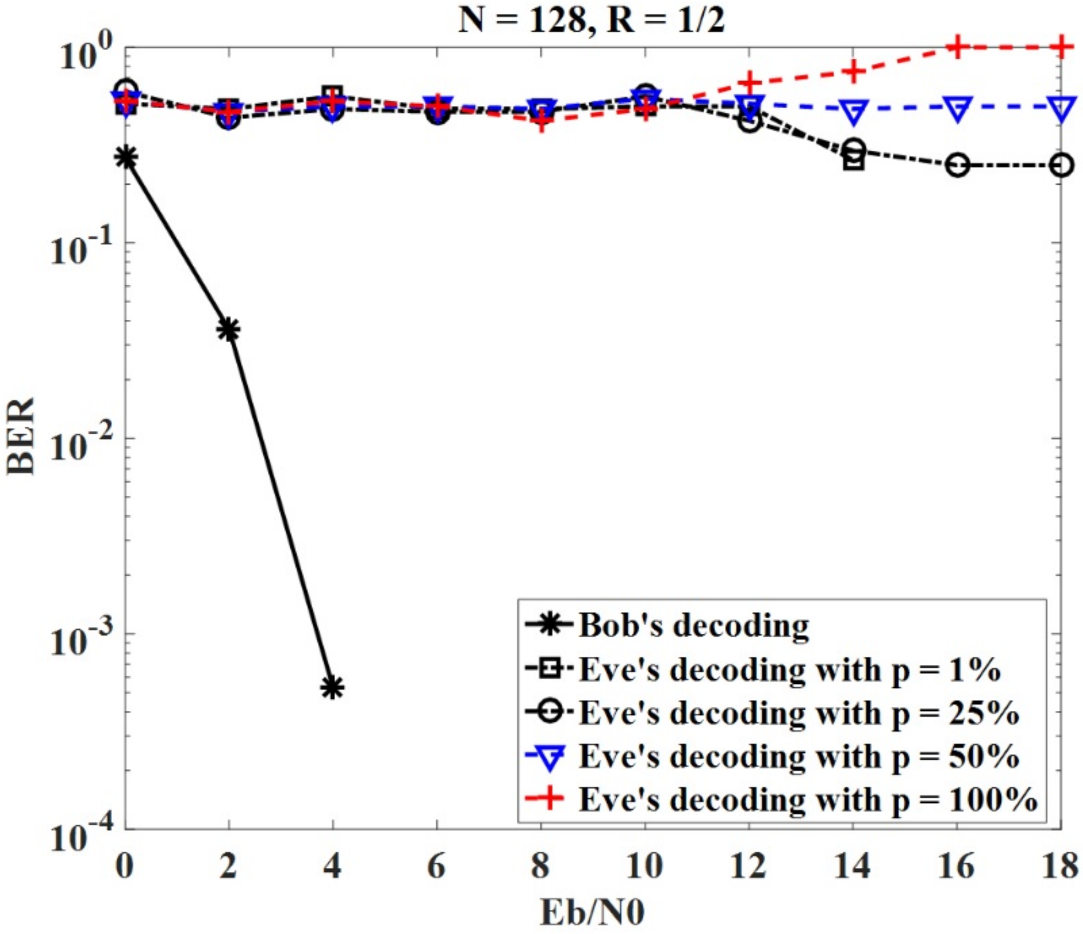

S 11 Fig. Security performance for $N = 256$, $R = 1/4$.

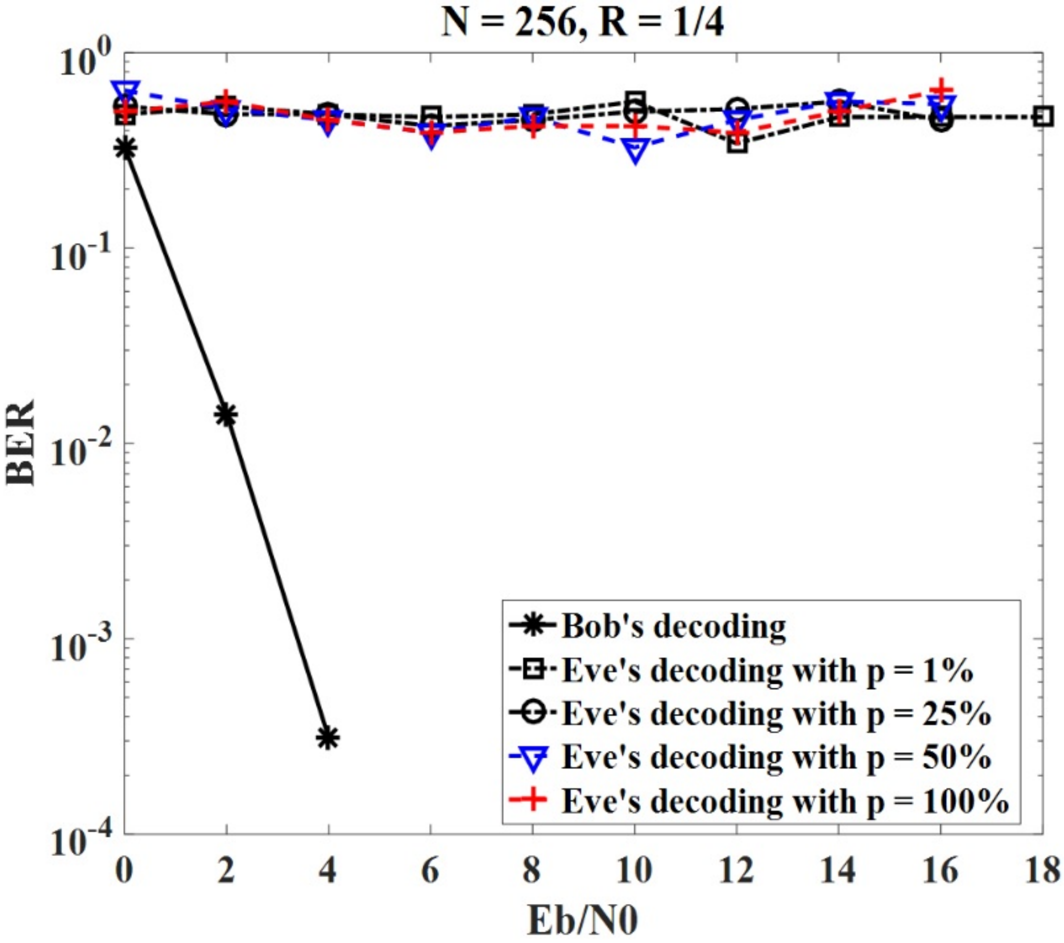

S 1 Table. Parameters for some NIST tests

NIST tests	Block length
Block frequency	128
Approximate entropy	10
Serial	4

S 2 Table. The tested values of the required NIST tests.

Test	128 bits	256 bits	512 bits	1024 bits	2048 bits
Monobit	1	1	1	1	1
Block Freq.	0.85	0.71	0.25	0.88	0.63
Runs	0.42	0.55	0.48	0.62	0.70
Longest 1s	0.49	0.92	0.63	0.78	0.38
DFT	0.76	0.20	0.36	0.41	0.28
Serial 1	0.56	0.61	0.45	0.77	0.33
Serial 2	0.23	0.78	0.43	0.52	0.82
Approx. Entropy	1	1	1	1	1
Cum. sums	0.98	0.83	0.72	0.95	0.85

S 3 Table. Simulation parameters.

Item	Specification
Code length (N)	128, 256, 512, 1024, 2048 bits
Key length	128, 256, 512, 1024, 2048 bits
Code rate (R)	1/2, 1/4, 1/8
Channel	AWGN
Decoding algorithm	SC
Key length	128, 256, 512, 1024, 2048 bits

S 4 Table. Comparing our method with the previous methods for $N = 1024$ and $R = 1/2$.

Techniques	BER at $E_b/N_0 = 2dB$
Ours	3×10^{-2}
[18]	$\approx 3 \times 10^{-2}$
[19]	$\approx 3 \times 10^{-2}$
[26]	$\approx 3 \times 10^{-2}$

S 5 Table. Comparing the security performance of the proposed method andthe previous methods.

Our method	BER of Eve at $E_b/N_0 = 10dB$
Our methods	0.5
[25]	$\approx 3 \times 10^{-3}$
[27]	$\approx 3 \times 10^{-3}$

S 6 Table. Total time complexity of the conventional polar code and the proposed secure polar code with $R = 1/2$.

Code length (bits)	128	256	512	1024	2048
Time complexity for conventional polar code	3.43	9.24	30.12	116.02	853.54
Time complexity for proposed polar code	3.38	9.11	30.70	116.05	853.17
