# Supplementary material for: Crypto-coding technique based on polar code and secret key generated from wireless channel characteristics for wireless communication systems
Source: PLoS One. 2025 Feb 25;20(2):e0318110. doi: 10.1371/journal.pone.0318110 (PMC12072636; doi:10.1371/journal.pone.0318110)
Supplement: Attachment — Submitted filename: 1.Response-to-Reviewers_R2.docx [file pone.0318110.s004.docx]

**Original Manuscript ID:** PONE-D-24-25066R1

**Original Article Title:** “Crypto-Coding Technique Based on Polar Code and Secret Key Generated from Wireless Channel Characteristics for Wireless Communication Systems”

**To:** PLOS ONE Editor

**Re:** Response to reviewers

Dear Editor,

The authors would like to thank the Editor and reviewers for their helpful comments on the manuscript. Based on the reviewers' comments, the manuscript has been carefully
revised from the previous submission. The detailed responses to the reviewers' comments
are given below.

Please note that the page, table, and figures may have been changed in the revised
manuscript.

To help with the legibility of this response letter, all the reviewers’
comments and questions are typeset in italic font and green color. Our answers are written in plain font. Rephrased sentences are typeset in ***blue*** color in this letter and are highlighted in the updated manuscript.

We are submitting two versions of the revised manuscript, a version with the
changes colored and a "clean" version.

Best regards,

Hoang Thi Phuong Thao et al.

**Reviewer#2, Concern # 1:** *Very unsatisfied with the literature review in the Introduction section.*

**Author answer:** Thank you for your comments. We rewrote the literature review in the Introduction section of the updated manuscript as follows:

Security and reliability are crucial factors for modern wireless communication systems [1]. They can be implemented at the physical and the upper layers which complement each other to ensure secure and reliable communication in systems. In terms of reliability, the upper layers use error correction and retransmission techniques, whereas the physical layer provides an important contribution to ensure accurate data delivery, which can employed by channel coding [2]. For security, they offer robust encryption mechanisms, but at a higher resource cost. In contrast, the physical layer security protects data with a lower resource consumption by relying on the physical characteristics of the communication channel [3]. Therefore, physical layer security has been receiving more attention from researchers [4]. Traditionally, data encryption and channel coding at the physical layer are two completely independent blocks in most secure wireless communication systems, which increases the computational complexity and latency [5]. To overcome these disadvantages, the crypto-coding technique that performs encryption and error correction in a single step has attracted significant attention in the last decade. Previous studies proposed crypto-coding techniques based on various channel coding such as low-density parity-check (LDPC) code for mobile 5G [6], Turbo code for software-defined radio systems, satellite and mobile (3G, 4G) [7–17], and polar code for 5G [18–30]. Polar codes, found by Arikan in 2009, are primarily utilized as channel code in 5G thanks to their low encoding and decoding complexity, high error correction performance, and flexible code rate [31]. The crypto-coding technique is performed by using secret keys, which can be generated from classical methods [18–25] or wireless channel characteristics [26–30], to control either the frozen bits or the information bits of polar code. However, the techniques using the secret keys generated from classical methods lead to an increase in computational complexity and require a complicated key management infrastructure. On the other hand, key generation from wireless channel characteristics is a potential alternative because of its lightweight and requirement of simpler hardware [32]. Therefore, the combination of polar codes and keys generated from wireless channel characteristics is a promising technology in the 5G system. In [26], the authors use a secret key extracted from channel state information (CSI) to assign frozen bits of polar code. The proposed technique provides the same bit error rate (BER) as the traditional method and a large key space. The work in [27] proposes a technique to improve physical layer security by using random channel gain of legal transmission to find out the frozen bit patterns. The investigation reported in [28], a frozen-bit pattern algorithm based on a channel quality indicator is developed to encrypt the transmitted message over the wireless channel, leading to a low block error rate (BLER). The authors in [29] measure channel entropy in real scenarios to extract the secret key. Then, the secret key is assigned into the frozen bits of the polar code. In [30], the authors use the random phases extracted from wireless channels as input chaotic sequence generators. To improve the security level, the authors add the Advanced Encryption Standard (AES) block to encrypt the generated chaotic sequence. The investigations in [26–30] have evaluated the reliability and security level by analyzing BER/BLER, or signal-to-noise ratio (SNR). However, these studies are limited in evaluating the error correction efficiency, computational complexity, and security level. Especially, they lack security evaluation according to the test of the National Institute of Standards and Technology (NIST) 800-22REV1A. Furthermore, it is worth noting that only a few studies on polar-based crypto-coding techniques using secret keys generated from wireless channel characteristics have been published in the open literature. Therefore, we believe this is an interesting topic to explore and a promising candidate to significantly enhance the reliability and security of 5G systems.

**Reviewer#2, Concern # 2:** *Not enough information for a reviewer to completely follow the experiments. Additional explanations to describe the experiment setup are required.*

**Author answer:** Thank you very much. We added more explanations for experiment setup in Section 3 – Simulation results of the updated manuscript as follows:

These keys are evaluated through 8 NIST tests [38] with some experimental parameters shown in Table 1. We need to set additional parameters of block length for NIST tests including Block frequency, Approximate entropy, and Serial. For the remaining five NIST tests, we only need to set the corresponding key length when running the results. The evaluation results are displayed in Table 2.

Table 1. Parameters for some NIST tests

| **NIST tests** | **Block length** |
| --- | --- |
| Block frequency | 128 |
| Approximate entropy | 10 |
| Serial | 4 |

Table 3. Simulation parameters

| **Item** | **Specification** |
| --- | --- |
| Code length (*N*) | 128, 256, 512, 1024, 2048 bits |
| Key length | 128, 256, 512, 1024, 2048 bits |
| Code rate (*R*) | 1/2, 1/4, 1/8 |
| Channel | AWGN |
| Decoding algorithm | SC |
| Modulation scheme | BPSK |

When running the simulations, the selected key length should correspond to the code length. For example, we choose key lengths of 128, 256, 512, 1024, and 2048 bits for code lengths of 128, 256, 512, 1024, and 2048 bits respectively. Additionally, for ease of representation in the figures, in different performance evaluations, we choose different key lengths/code lengths and code rates as follows:

- For evaluating the error correction ability of the proposed method:
- In case study 1: the code lengths and key lengths of 1024 bits and 2048 bits are unchanged in each situation. Then, we modify the code rates of 1/2 and 1/4.
- In case study 2: The code rates of 1/4 and 1/8 are unchanged in each situation. Then, we modify the code lengths and key lengths of 128 bits and 512 bits.
- For evaluating the security performance, we simulate a code length/key length of 128 bits and code rate of 1/2, and a code length/key length of 256 bits and code rate of 1/4.
- For evaluating computational complexity, we implement for all code lengths/key lengths of 128, 256, 512, 1024, and 2048 bits with a code rate of 1/2.

**Reviewer#2, Concern # 3:** *More technical comparisons need to be given with an existing one.*

**Author answer:** Thank you for your valuable feedback. We added more comparisons with the existing methods in the subsection of Security performance in the updated manuscript as follows:

To compare the security performance of the proposed method with the previous methods, we can employ the BER of Eve. Table 5 indicates the comparison results between our proposed method and the methods in [25] and [27]. It can be seen that our proposed method gives Eve’s BER almost unchanged, and is represented on the figure as a straight line. Meanwhile, Eve’s BER in studies in [25] and [27] decreases with increasing $E_{b}/N_{0}.$ For dB, BER values are 0.5 for our method, and around 10-3 for [25] and [27], respectively. Therefore, the methods in [25] and [27] reduce Eve’s decoding ability less than our method. It can be concluded that the security performance of the proposed method outperforms the existing methods in [25] and [27].

Table 5. Comparing the security performance of the proposed method and the previous methods

| **Method** | **BER of Eve at** **dB** |
| --- | --- |
| Our method | 0.5 |
| [25] | $\approx3\times10^{-3}$ |
| [27] | $\approx3\times10^{-3}$ |

**Reviewer#2, Concern # 4:** *The article does not address other threats, such as active attacks, side-channel attacks.*

**Author answer:** Thank you for your comment. In this work, we only consider the passive attacks to secure the data over the wireless transmission channel. In the future, we will test the proposed algorithm for active attack types. In the updated manuscript, we added more future work as follows:

Additionally, we will also implement the proposed method in the hardware structure and try more active attacks and realistic scenarios to verify its feasibility.

**Reviewer#2, Concern # 5:** *Using wireless channel may not always be secure. What is the threat model?*

**Author answer:** Thank you for your comment. In this research, we only consider the eavesdropping channel model with passive eavesdropping attack. The channel model is shown in Figure 1 of Section 2 – Materials and Methods.

**Reviewer#2, Concern # 6:** *It is not accurate to say that the method does not require additional hardware costs. Because relying on the characteristics of the wireless channel to generate and distribute keys may require additional software or firmware changes.*

**Author answer:** Thank you for your comment. In our last manuscript, we only mentioned that our proposed method does not require additional hardware overhead for key management and distribution, it still need hardware for orther tasks. Since the secret key generation techniques based on the wireless channel characteristics are lightweight, they do not require any support from devices for the key distribution and management process [32]. In the updated manuscript, we wrire more clealy as follows:

However, the techniques using the secret keys generated from classical methods lead to an increase in computational complexity and require a complicated key management infrastructure. On the other hand, key generation from wireless channel characteristics is a potential alternative because of its lightweight and requirement of simpler hardware [32]

[32]	J. Zhang, T. Q. Duong, A. Marshall, and R. Woods, “Key Generation from Wireless Channels: A Review,” *IEEE Access*, vol. 4, pp. 614–626, 2016, doi: 10.1109/ACCESS.2016.2521718.

**Reviewer#2, Concern # 7:** *We may need to evaluate the performance of the method under more realistic scenarios to verify its practical feasibility.*

**Author answer:** Thank you for your advice. In this study, we have only simulated the proposed method on MATLAB software. In the future, we will implement this proposed method on the hardware structure and try more realistic scenarios to verify its feasibility. In the updated manuscript, we added more future work as follows:

Additionally, we will also implement the proposed method in the hardware structure and try more active attacks and realistic scenarios to verify its feasibility.

**Reviewer#2, Concern # 8:** *The model has not been tested under other conditions, such as different code lengths, key lengths, or channel conditions.*

**Author answer:** Thank you for your feedback. In this research, the simulations are carried out in different key lengths, code lengths, and code rates through the AWGN channels. The obtained results are shown in the Section 3-Simulation results.
