# Supplementary material for: Crypto-coding technique based on polar code and secret key generated from wireless channel characteristics for wireless communication systems
Source: PLoS One. 2025 Feb 25;20(2):e0318110. doi: 10.1371/journal.pone.0318110 (PMC12072636; doi:10.1371/journal.pone.0318110)
Supplement: Attachment — Submitted filename: Review.docx [file pone.0318110.s002.docx]

Reviewer Comments

The authors in this article proposed a technique Crypto-Coding Technique Based on Polar Code and Secret Key Generated from Wireless Channel Characteristics for Wireless Communication Systems.

 The following feedback is required to incorporate for improving the content of the paper in further:

1. Please ensure that the paragraph is well-developed, coherent, concise, and can stand alone as a unit of information. It should cover all essential academic elements of the full-length paper, including the background, purpose, focus, methods, results, and conclusions.
2. Please review your mathematical equations once according to your paper design. Presentation of equations is somewhat ambiguous need to incorporate
3. Enhance your literature by including every technique that you used in your paper.
4. Add a summary paragraph at the end of Material and Method.
5. Add a summary paragraph at the end of results section presents your results in abstract form.
6. Add comparative analysis and critical analysis of the work in Results section.
7. Clearly highlights the gaps and future work in conclusion section.
8. The English of the paper should be polished further.

**Resubmission**
