# Supplementary material for: Crypto-coding technique based on polar code and secret key generated from wireless channel characteristics for wireless communication systems
Source: PLoS One. 2025 Feb 25;20(2):e0318110. doi: 10.1371/journal.pone.0318110 (PMC12072636; doi:10.1371/journal.pone.0318110)
Supplement: Attachment — Submitted filename: Response to Reviewers.docx [file pone.0318110.s003.docx]

**Original Manuscript ID:** PONE-D-24-25066

**Original Article Title:** “Crypto-Coding Technique Based on Polar Code and Secret Key Generated from Wireless Channel Characteristics for Wireless Communication Systems”

**To:** PLOS ONE Editor

**Re:** Response to reviewers

Dear Editor,

The authors would like to thank the Editor and reviewers for their helpful comments on the manuscript. Based on the reviewers' comments, the manuscript has been carefully
revised from the previous submission. The detailed responses to the reviewers' comments
are given below.

Please note that the page, table, and figures may have been changed in the revised
manuscript.

To help with the legibility of this response letter, all the reviewers’
comments and questions are typeset in italic font and green color. Our answers are written in plain font. Rephrased sentences are typeset in ***blue*** color in this letter and are highlighted in the updated manuscript.

We are submitting two versions of the revised manuscript, a version with the
changes colored and a "clean" version.

Best regards,

Hoang Thi Phuong Thao et al.

**Editor, Concern # 1:** *The Authors not properly follow the PLOS ONE Paper format.*

**Author answer:** Thank you for your suggestion. The updated manuscript has been revised to follow the PLOS ONE Paper format.

**Editor, Concern # 2:** *The authors should rewrite the abstract in a concise form.*

**Author answer:** Thank you for your comment. We rewrote the abstract in the updated manuscript as below:

The crypto-coding technique is essential for modern digital wireless communications systems, allowing data encryption and channel encryption to be performed in only one step without requiring additional hardware. This work proposes a crypto-coding technique combining polar codes with a secret key, which is derived from wireless channel characteristics, to boost the security and reliability characteristics of the systems. The secret key is divided into two parts, one is assigned to the frozen bits, and the other is XORed with the information bits. A simulation with different key lengths and code lengths is carried out in Additive white Gaussian noise (AWGN) that helps us to evaluate this technique by the error correction and security performance, computational complexity. The key benefit is that it achieves the same bit error rate (BER) performance and computational complexity as traditional polar codes and existing methods despite only taking one step. Meanwhile, it ensures completely degraded decoding effectiveness at eavesdroppers, thus it is effective against passive eavesdropping attacks. Furthermore, this method does not require additional hardware overhead for key management and distribution.

**Editor, Concern # 3:** *Literature is not presented in desirable ways.*

**Author answer:** Thank you for your advice. We rewrote the literature in the updated manuscript as follows:

Security and reliability are crucial factors for fifth-generation (5G) wireless communication systems [1]. To ensure data security, information security methods often use classical encryption methods at the upper layers. However, due to limited resources such as CPU, power, and storage size, e.g., wireless communication devices face many difficulties in integrating these traditional encryption methods [2]. Meanwhile, reliability is performed at the physical layer by utilizing some channel coding techniques, such as Turbo code, low-density parity-check (LDPC) code, and Polar code [3]. Traditionally, data encryption and channel coding are two completely independent blocks in most secure wireless communication systems, increasing the computational complexity and latency [4]. To overcome these disadvantages, the crypto-coding technique that performs encryption and error correction in a single step has attracted significant attention in the last decade. Some crypto-coding techniques for satellite and fourth-generation (4G) communication systems are based on Turbo code [4]–[14].

For the 5G communication systems, the polar code found by Arikan in 2009 [15] has been used as the channel code with many advantages, such as Low Encoding and Decoding Complexity, high error correction performance, and flexible code rate, …. Existing studies of crypto-coding techniques based on polar codes use secret keys to control either the frozen bits or the information bits. The secret keys can be generated from third parties [16]–[22] or wireless channel characteristics [23]–[26]. The techniques using the secret keys generated from third parties lead to an increase in computational complexity and require hardware structure for key distribution and management, thus they are not suitable for 5G communication systems.

In recent years, key generation from wireless channel characteristics is a potential alternative to third-party key generation because it is lightweight and does not require additional hardware from the device [27]. As a result, the combination of polar codes and keys generated from wireless channel characteristics will reduce complexity and delay in the 5G system. In [23], the authors use a secret key extracted from channel state information (CSI) to assign frozen bits of polar code. However, this work lacks an evaluation of the randomness of the secret key and the security level of the proposed secure polar code. In [24], Yang *et. al*. propose a technique to improve physical layer security by using random channel gain of legal transmission to find out the frozen bit patterns. The investigation reported in [25], a frozen-bit pattern algorithm based on a channel quality indicator is developed to encrypt the transmitted message over the wireless channel. Another method in [26] also applies the secret keys to fill in the frozen bits, but it does not need CSI from any network channels. Nevertheless, the investigations in [24]–[26] only evaluate security level by comparing the bit error rates (BERs) or block error rates (BLERs) of the legitimate receiver (Bob) and the eavesdropper (Eve) without evaluating the complexity or effectiveness of the proposed methods compared to traditional polar code. In summary, there are still few studies on the technique that uses secret keys generated from the wireless channel characteristics to interfere with the components of polar codes. The current studies are limited in evaluating the error correction efficiency, computational complexity, and security level.

**Editor, Concern # 4:**  *The whole paper flow control and connectivity is ambiguous, it is difficult for readers.*

**Author answer:** Thank you for your valuable suggestion. In the updated manuscript, we rearranged the flow control as follows:

Abstract

1. Introduction
2. Materials and Methods
   2.1. Secret key generation from wireless channel characteristics
   2.2. Polar code
   2.3. Proposed secure polar code
3. Results and Discussion
   3.1. Evaluation of the error correction ability of the proposed method
   3.2. Security performance
   3.3. Computational complexity
4. Conclusions

References

**Editor, Concern # 5:**  *Make sure the paper format in terms of Introduction, Literature, Proposed Methodology, and Results and Discussion etc.*

**Author answer:** Thank you for your comment. The update manuscript has revised follow the PLOS ONE Paper format.

**Editor, Concern # 6:** *Conduct some critical analysis to prove the strength of the proposed scheme*

**Author answer:** Thank you for your suggestion. We added more analysis for our proposed scheme on the 5^th^ and 6^th^ page of the updated manuscript as follows:

For the conventional polar code, the frozen bits are wasted and set up by zero sequences. In this proposed method, the second secret key part *K*_2_ is assigned in the frozen bits$u_{A^{c}}.$ This makes it impossible for attackers to guess the frozen values. At this time, the frozen bits will be:

$u_{A^{c}}=K_{2}$ (9)

In this way, both information bits and frozen bits are kept secret by using the secret key *K*.

As shown in Figure 4, the decoding process corresponds to the encoding process. It is assumed that the legal receiver generates the same key *K* as the transmitter, thus the same *K*_1_ and *K*_2_ parts are divided.

In this proposed method, the signal is randomly processed after passing the channel coding module in the physical layer. This makes the code word after the encoding process confusing. This method not only assures the error correction and encryption functions but also utilizes the physical layer's wireless channel property for secret key extraction.

**Editor, Concern # 7:** *Figures 4 and 5 are not well presented, blured need to explain in concise manner to show the scope of the research*

**Author answer:** Thank you for your comment. We fixed representation errors in Figures 4 and 5 in the updated manuscript.

**Editor, Concern # 8:** *Add some comparative analysis with your method.*

**Author answer:** Thank you for your suggestion. We added more analysis on the 8^th^ and 9^th^ page of the simulation results section as follows:

It can be seen that the time complexity of our proposed method is close to that of the conventional polar code and it increases as code length increases.

All methods give the complexity of $O\left(N\log N\right)$for both encoding and decoding processes. Consequently, the computational complexity of our method is equivalent to that of the previous methods.

Overall, the proposed crypto-coding does not change the error correction efficiency and computational complexity compared with the conventional polar code. In addition, it also increases the data confidentiality when transmitted over the wireless channel.

**Editor, Concern # 9:** *Clearly highlights the gaps and future work in conclusion section.*

**Author answer:** Thank you for your valuable comment. We added more gaps and future work in the conclusion section on the 9^th^ page of the updated manuscript as below:

Until now, we only focused on a single-antenna channel model. In the future, we will extend this research to the multiple-input multiple-output (MIMO) systems, and other advanced channel codes can be considered.

**Editor, Concern # 10:** *The English of the paper should be polished further.*

**Author answer:** Thank you for your suggestion. We corrected some grammar errors and sentences in the updated manuscript.

**Reviewer#1, Concern # 1:** *Please ensure that the paragraph is well-developed, coherent, concise, and can stand alone as a unit of information. It should cover all essential academic elements of the full-length paper, including the background, purpose, focus, methods, results, and conclusions.*

**Author answer:** Thank you for your comments. We have revised the manuscript according to your comments to address this concern and enhance the paper’s quality. Specifically, we improved the introduction, strengthened the comparative analysis, and restructured the paper as highlighted in the updated manuscript .

**Reviewer#1, Concern # 2:** *Please review your mathematical equations once according to your paper design. Presentation of equations is somewhat ambiguous need to incorporate*

**Author answer:** Thank you very much. We rewrote some mathematical equations in the updated manuscript as follows:

$\widehat{u}_{i}\triangleq\left\{\begin{matrix} u_{i},\begin{matrix} & \mathrm{if}\begin{matrix} & i\in A^{c} \end{matrix} \end{matrix} \\ h_{i}\left(y_{1}^{N},\widehat{u}_{1}^{i-1}\right),\begin{matrix} & \mathrm{if}\begin{matrix} & i\in A \end{matrix} \end{matrix} \end{matrix}\right.$ (6)

$h_{i}\left(y_{1}^{N},\widehat{u}_{1}^{i-1}\right)\triangleq\left\{\begin{matrix} 0,\begin{matrix} & L_{N}^{i}\left(y_{1}^{N},\widehat{u}_{1}^{i-1}\right)\geq1 \end{matrix} \\ 1,\begin{matrix} & otherwise \end{matrix} \end{matrix}\right.$ (7)

**Reviewer#1, Concern # 3:** *Enhance your literature by including every technique that you used in your paper.*

**Author answer:** Thank you for your valuable feedback. We have impoved the literature in Introduction Section as follows:

Security and reliability are crucial factors for fifth-generation (5G) wireless communication systems [1]. To ensure data security, information security methods often use classical encryption methods at the upper layers. However, due to limited resources such as CPU, power, and storage size, e.g., wireless communication devices face many difficulties in integrating these traditional encryption methods [2]. Meanwhile, reliability is performed at the physical layer by utilizing some channel coding techniques, such as Turbo code, low-density parity-check (LDPC) code, and Polar code [3]. Traditionally, data encryption and channel coding are two completely independent blocks in most secure wireless communication systems, increasing the computational complexity and latency [4]. To overcome these disadvantages, the crypto-coding technique that performs encryption and error correction in a single step has attracted significant attention in the last decade. Some crypto-coding techniques for satellite and fourth-generation (4G) communication systems are based on Turbo code [4]–[14].

For the 5G communication systems, the polar code found by Arikan in 2009 [15] has been used as the channel code with many advantages, such as Low Encoding and Decoding Complexity, high error correction performance, and flexible code rate, …. Existing studies of crypto-coding techniques based on polar codes use secret keys to control either the frozen bits or the information bits. The secret keys can be generated from third parties [16]–[22] or wireless channel characteristics [23]–[26]. The techniques using the secret keys generated from third parties lead to an increase in computational complexity and require hardware structure for key distribution and management, thus they are not suitable for 5G communication systems.

In recent years, key generation from wireless channel characteristics is a potential alternative to third-party key generation because it is lightweight and does not require additional hardware from the device [27]. As a result, the combination of polar codes and keys generated from wireless channel characteristics will reduce complexity and delay in the 5G system. In [23], the authors use a secret key extracted from channel state information (CSI) to assign frozen bits of polar code. However, this work lacks an evaluation of the randomness of the secret key and the security level of the proposed secure polar code. In [24], Yang *et. al*. propose a technique to improve physical layer security by using random channel gain of legal transmission to find out the frozen bit patterns. The investigation reported in [25], a frozen-bit pattern algorithm based on a channel quality indicator is developed to encrypt the transmitted message over the wireless channel. Another method in [26] also applies the secret keys to fill in the frozen bits, but it does not need CSI from any network channels. Nevertheless, the investigations in [24]–[26] only evaluate security level by comparing the bit error rates (BERs) or block error rates (BLERs) of the legitimate receiver (Bob) and the eavesdropper (Eve) without evaluating the complexity or effectiveness of the proposed methods compared to traditional polar code. In summary, there are still few studies on technique that uses secret key generated from the wireless channel characteristics to interfere with the components of polar codes. The current studies are limited in evaluating the error correction efficiency, computational complexity and security level.

**Reviewer#1, Concern # 4:** *Add a summary paragraph at the end of Material and Method.*

**Author answer:** Thank you very much. We added more a summary paragraph at the end of Material and Method Section in the 6^th^ page of the updated manuscript as below:

Consequently, This method uses a key generated from the radio channel to control the components of the polar code, allowing it to save hardware configuration while still ensuring simultaneous implementation of error correction and encryption functions.

**Reviewer#1, Concern # 5:** *Add a summary paragraph at the end of results section presents your results in abstract form.*

**Author answer:** Thank you for your comment. We added a summary paragraph at the end of Section – Simulation results in the 9^th^ page of the updated manuscript as follows:

Overall, the proposed crypto-coding does not change the error correction efficiency and computational complexity compared with the conventional polar code. In addition, it also increases the data confidentiality when transmitted over the wireless channel.

**Reviewer#1, Concern # 6:** *Add comparative analysis and critical analysis of the work in Results section.*

**Author answer:** Thank you for your comment. We added more analysis in the 9^th^ page of simulation results section as follows:

It can be seen that the time complexity of our proposed method is close to that of the conventional polar code and it increases as code length increases. For instance, the time complexities of both methods offer approximate values of 3, 9, 30, 116, and 853 for the key lengths of 128, 26, 512, 1024, and 2048, respectively.

All methods give the complexity of $O\left(N\log N\right)$for both encoding and decoding processes. Consequently, the computational complexity of our method is equivalent to that of the previous methods.

**Reviewer#1, Concern # 7:** *Clearly highlights the gaps and future work in conclusion section.*

**Author answer:** Thank you for your advice. We added more gaps and future work in the conclusion section on the 9^th^ page of the updated manuscript as below:

Until now, we only focused on a single-antenna channel model. In the future, we will extend this research to the massive multiple-input multiple-output (MIMO) systems, and other advanced channel codes can be considered.

**Reviewer#1, Concern # 8:** *The English of the paper should be polished further.*

**Author answer:** Thank you for your feedback. We have corrected some grammar errors and refined sentences in the updated manuscript.
